# Modification of Functional, Antioxidant, and Anti‐Allergic Properties of Fermented Silkworm Pupae Protein During In Vitro Gastrointestinal Digestion

**DOI:** 10.1002/fsn3.72248

**Published:** 2026-08-26

**Authors:** Jing Yang, Linqing Zhang, Shuling Zhou, Sutee Wangtueai, Jiajia Song

**Affiliations:** ^1^ School of Food Science and Engineering Chongqing Technology and Business University Chongqing China; ^2^ Faculty of Agro‐Industry Chiang Mai University Chiang Mai Thailand; ^3^ College of Food Science Southwest University Chongqing China

**Keywords:** allergenicity reduction, antioxidant activity, functional properties, lactic acid bacteria fermentation, silkworm pupae protein

## Abstract

Silkworm pupae protein (SPP) is a promising alternative protein, but its application is limited by allergenicity and suboptimal functionality. Here, SPP was fermented with *Lactiplantibacillus plantarum* Z4 and subjected to in vitro gastrointestinal digestion to evaluate the stability and bioaccessibility of its functional properties. Fermentation increased the degree of hydrolysis, α‐helix content, surface hydrophobicity, and free sulfhydryl content, indicating structural rearrangement. Emulsion stability was improved, whereas foaming capacity, foam stability, and emulsifying activity were unchanged. Fermentation also enhanced antioxidant activity and reduced allergenicity‐related responses. After digestion, both unfermented and fermented samples showed higher antioxidant activity and lower allergenicity‐related responses, while fermented digests generally showed greater antioxidant activity and lower IgE‐inhibition activity than the corresponding unfermented digests. These findings suggest that Z4 fermentation acts as a pre‐digestion modification strategy that improves SPP functionality, and gastrointestinal digestion provides a physiologically relevant evaluation of the bioaccessibility of its functional properties.

## Graphical abstract

*Lactiplantibacillus plantarum* Z4 fermentation modifies the structural and functional properties of silkworm pupae protein, improves its foaming/emulsion stability and antioxidant capacity, and reduces its IgE‐binding potential following simulated gastrointestinal digestion.

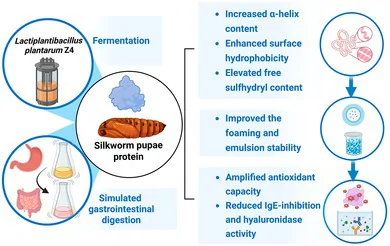

## 1 Introduction

Silkworm pupae represent a protein‐rich edible insect resource with a well‐balanced amino acid composition, offering high nutritional value and considerable potential for use in food and functional ingredient development (Yang, Zhou, et al. 2025; Yang et al. 2023). Studies have demonstrated that silkworm pupae protein (SPP) possesses not only excellent nutritional qualities but also diverse bioactive functions, including antioxidant, hypoglycemic, hypolipidemic, and immunomodulatory activities (He et al. 2021; Yang et al. 2023). However, the native structure of SPP is characterized by dense protein packing and a high degree of α‐helix and β‐sheet aggregation, leading to poor solubility as well as limited emulsifying and foaming properties, thereby restricting its applicability in food processing (Yang, Zhou, et al. 2025). More critically, several immunogenic and allergenic proteins have been identified in SPP, which can bind to IgE antibodies and trigger a range of allergic reactions, including asthma, urticaria, allergic rhinitis, and even anaphylaxis (He et al. 2021). This potential allergenicity poses a significant safety concern for consumers and remains a major obstacle to the broader application of SPP in the food industry. Therefore, developing a green, safe, and controllable strategy to reduce the allergenicity of SPP is of great practical importance.

Lactic acid bacteria (LAB) fermentation is a natural modification technique widely applied in the food industry, offering unique advantages in protein modification. It can regulate protein conformation and improve functional properties such as emulsifying, foaming, and antioxidant activities (Cao et al. 2019; Jiang et al. 2020). For example, fermentation with *Lactiplantibacillus plantarum* and *Bacillus subtilis natto* enhances the emulsifying properties of peanut protein (Pi et al. 2022), while *Lactobacillus helveticus* fermentation improves the foaming capacity of soybean protein (Meinlschmidt et al. 2016). LAB fermentation also increases antioxidant activity and structural flexibility in plant proteins such as millet and pea (Emkani et al. 2022; Yakubu et al. 2022). Similarly, fermentation of edible insect flours (mealworm and grasshopper) with *Lactococcus lactis* increases peptide diversity and antioxidant activity (Mendoza‐Salazar et al. 2021). Importantly, gastrointestinal digestion provides a physiologically relevant means to evaluate the stability and bioaccessibility of these effects. The in vitro digestion markedly enhanced antioxidant activities of baked insects ( *Gryllodes sigillatus* , *Tenebrio molitor* , and *Schistocerca gregaria*) (Zielińska et al. 2017, 2018). Likewise, enzymic hydrolysates of SPP, mimicking digestive processes, exhibited strong antioxidant effects in HepG2 cells (Cermeño et al. 2022). Therefore, we hypothesize that fermentation‐induced structural modifications of SPP may affect the functional properties and bioactivities of its digests.

In addition, LAB fermentation is recognized as an effective natural strategy for modulating protein structure and immunoreactivity. During fermentation, proteolytic enzymes produced by microorganisms degrade intact proteins into smaller peptides, potentially disrupting IgE‐binding epitopes and thereby reducing allergenicity (Fu et al. 2023; Lu et al. 2022; Pi et al. 2022). Numerous studies have demonstrated that LAB fermentation can significantly reduce the IgE‐binding capacity of highly allergenic proteins such as peanut, wheat, and soybean (Fu et al. 2023; Pi et al. 2022; Xing et al. 2024). This reduction is generally attributed to structural modifications during fermentation, including partial hydrolysis, peptide fragmentation, and changes in protein conformation that mask or destroy IgE‐binding epitopes. Furthermore, in vitro simulated digestion often leads to additional decreases in allergenicity (Wang et al. 2021; Yang et al. 2024), as the combined effects of microbial enzymes and digestive proteases further break down allergenic epitopes and generate smaller peptides with lower immunoreactivity.

Although LAB fermentation has been widely applied to improve protein functionality and reduce allergenicity in plant and insect proteins, little is known about its effects on SPP. SPP possesses unique structural and nutritional features that may respond differently to microbial modification. Previous studies have rarely investigated how fermentation‐driven structural alterations, combined with gastrointestinal digestion, influence both the functional and immunological properties of SPP. In this study, *Lactiplantibacillus plantarum* Z4, a protease‐active strain isolated from fermented cowpea, was employed to enhance SPP functionality and reduce allergenicity. Therefore, this study was designed to distinguish between two related but different effects: the direct modification of SPP induced by Z4 fermentation before digestion, and the stability and bioaccessibility of the resulting functional and anti‐allergic properties during simulated gastrointestinal digestion. This distinction allows us to evaluate whether fermentation provides additional benefits beyond those generated by digestive proteolysis alone.

## 2 Materials and Methods

### 2.1 Materials and Reagents

Fresh silkworm pupae were purchased from Jintang County, Chengdu, Sichuan Province, China. 8‐Anilino‐1‐naphthalenesulfonic acid (ANS, A1028) and hyaluronidase (H3506) were obtained from Sigma‐Aldrich (St. Louis, MO, USA). Immunological reagents including goat anti‐mouse IgE secondary antibody (SA5‐10262) and horseradish peroxidase (HRP)‐conjugated antibody (SA5‐10263) were sourced from Thermo Fisher Scientific (Cleveland, OH, USA). Pepsin (P8160) and trypsin (T8150) were purchased from Solarbio Science & Technology Co. Ltd. (Beijing, China). The total antioxidant capacity assay kit (FRAP method, S0116) was obtained from Beyotime Biotechnology (Shanghai, China). Other reagents used in this study included: n‐hexane (analytical grade, for defatting), 1 M NaOH and 1 M HCl (for pH adjustment), KCl, KH_2_PO_4_, NaHCO_3_, NaCl, MgCl_2_·6H_2_O, and (NH_4_)_2_CO_3_ (for preparation of simulated gastric and intestinal fluids), CaCl_2_, porcine bile salts, SDS (sodium dodecyl sulfate), OPA reagent (for hydrolysis analysis), DPPH, ABTS, potassium persulfate (for ABTS radical generation), Ellman's reagent (DTNB), glycine buffer, 0.01 M phosphate buffer (pH 7.0), TMB substrate solution, Ehrlich's reagent. All chemicals were of analytical grade unless otherwise specified.

### 2.2 Preparation of Defatted Silkworm Pupae Powder

Fresh pupae of silkworms were lyophilized and ground into fine powder. A defatting step was performed by blending the powder with *n*‐hexane (solid‐to‐solvent ratio of 1:5, w/v), followed by ultrasonic treatment (40 kHz, 120 W, 25°C) for 30 min. The mixture was then centrifuged at 3000 × *g* for 10 min, and the resulting upper phase was removed. This procedure was carried out four times to ensure effective lipid removal. The remaining material was left to dry under ventilation overnight, yielding the defatted silkworm pupae powder (Yang, Zhou, et al. 2025).

### 2.3 Preparation of Silkworm Pupae Protein

According to Yang, Zhou, et al. (2025), defatted pupae powder was mixed with distilled water at a ratio of 1:10 (w/v), and the pH was adjusted to 9.0 using 1 M NaOH. The suspension was stirred at 25°C for 4 h while maintaining the pH at 9.0. After centrifugation at 5000 × *g* for 10 min, the supernatant was collected and acidified to pH 4.5 using 1 M HCl to induce isoelectric precipitation. The precipitate was collected by centrifugation (5000 × *g*, 10 min), neutralized to pH 7.0, dialyzed for 24 h, and freeze‐dried to obtain SPP powder.

### 2.4 Culture and Morphological Observation of Lactic Acid Bacteria (LAB)

*Lactiplantibacillus plantarum* Z4, a strain isolated from traditionally fermented cowpea, was inoculated at 2% (v/v, 200 μL) into 10 mL of MRS broth and incubated statically at 37°C for 24 h. Two serial subcultures were performed. Colonies were streaked onto MRS agar and incubated at 37°C for 48 h. The isolate used in this study was preliminarily characterized based on colony and cell morphology. Its identity was further confirmed by 16S rDNA gene sequencing using universal primers, followed by sequence homology analysis against the NCBI database. Detailed procedures and phylogenetic analysis are provided in the Supporting Informations.

### 2.5 Determination of Protease Activity of *Lactiplantibacillus* *plantarum* Z4

The protease activity of *Lactiplantibacillus plantarum* Z4 was evaluated by the agar well diffusion method (Kuerman et al. 2024). Skim milk agar medium was prepared, and wells were made in the solidified agar. An aliquot of the culture supernatant of strain Z4 was added into each well, and the plates were incubated at 37°C for 24 h. Protease activity was assessed based on the formation of a clear hydrolysis zone around the well. The diameter of the hydrolysis zone was measured to reflect the proteolytic activity of strain Z4.

### 2.6 Fermentation of Silkworm Pupae Protein

Fermentation was conducted according to Xiang et al. (2023). SPP was mixed with sterile water (1:20, w/v), sealed, and heat‐treated at 85°C for 15 min. Bacterial cultures were centrifuged (8000 × *g*, 4°C, 10 min), resuspended in 0.9% NaCl, and washed twice. The cooled protein solution was inoculated with 10% LAB (10^8^ CFU/mL) and fermented at 37°C, 180 rpm for 48 h. The fermented sample was designated as FSPP and labeled as Z4 in figures comparing fermented and non‐fermented samples, while the non‐fermented control, prepared by replacing the LAB inoculum with sterile saline, was designated as SPP and labeled as CON.

### 2.7 In Vitro Simulated Gastrointestinal Digestion

Simulated gastric digestion was performed according to He et al. (2021), with minor modifications. Both SPP and FSPP were subjected to identical gastric and intestinal digestion procedures.

For gastric digestion, SPP or FSPP solution (50 mg/mL) was mixed with pre‐warmed 1.25× simulated gastric fluid (SGF, pH 6.16) at a ratio of 1:1 (v/v). The SGF contained 8.63 mM KCl, 1.13 mM KH_2_PO_4_, 31.25 mM NaHCO_3_, 59.00 mM NaCl, 0.15 mM MgCl_2_·6H_2_O, and 0.63 mM (NH_4_)_2_CO_3_. The pH was adjusted to 3.0 ± 0.1 with 1 mol/L HCl, followed by the addition of CaCl_2_ to a final concentration of 0.075 mmol/L and pepsin to a final activity of 600 U/mL. The mixture was incubated at 37°C and 300 rpm for 2 h, and the reaction was terminated by adjusting the pH to 7.0 ± 0.1 with 1 mol/L NaOH. After centrifugation at 7500 × g for 15 min, the supernatants were collected and stored at −80°C. The gastric digests were designated as SPP‐SGF and FSPP‐SGF, respectively.

For intestinal digestion, the gastric digest was mixed with pre‐warmed 1.25× simulated intestinal fluid (SIF, pH 7.2) at a ratio of 1:1 (v/v). The 1.25× SIF contained 8.50 mM KCl, 1.00 mM KH_2_PO_4_, 106.25 mM NaHCO_3_, 48.00 mM NaCl, and 0.41 mM MgCl_2_·6H_2_O. The pH was adjusted to 7.0 ± 0.1 with 1 mol/L NaOH, followed by the addition of porcine bile salts, CaCl_2_, and trypsin to final concentrations or activity of 10 mmol/L, 0.3 mmol/L, and 100 U/mL, respectively. The mixture was incubated at 37°C and 300 rpm for 2 h, and the reaction was terminated by heating at 95°C for 5 min. After centrifugation at 7500 × g for 15 min, the supernatants were collected and stored at −80°C. The intestinal digests were designated as SPP‐SIF and FSPP‐SIF, respectively.

### 2.8 SDS‐PAGE Analysis

The protein profile changes of SPP and FSPP before and after in vitro gastrointestinal digestion were analyzed using SDS‐PAGE according to the method of Laemmli (1970), with minor modifications, as described previously by He et al. (2021). Briefly, 80 μL of sample solution was mixed with 20 μL of 5× loading buffer, and the mixture was heated at 100°C for 5 min to denature the proteins. After cooling to room temperature, 10 μL of the prepared sample was loaded onto an SDS‐PAGE gel consisting of a 12% resolving gel and a 5% stacking gel. Electrophoresis was first conducted at 80 V for 20 min to allow proteins to migrate through the stacking gel, followed by 110 V for 60 min for protein separation in the resolving gel. After electrophoresis, the gel was stained with Coomassie Brilliant Blue R‐250 for 1 h at room temperature and then destained overnight in an acetic acid‐containing destaining solution until clear protein bands were visible.

### 2.9 Circular Dichroism (CD) Spectroscopy

CD spectra were recorded to assess secondary structure. SPP and FSPP solutions, corresponding to the CON and Z4 groups, respectively, were prepared in distilled water at 0.2 mg/mL. Spectra were acquired from 190 to 260 nm at 25°C using a CD spectrometer (Chirascan, Applied Photophysics, UK) with a 1.0 nm bandwidth and a scan rate of 100 nm/min. Secondary structure compositions (α‐helix, β‐sheet, β‐turn, random coil) were calculated using DichroWeb.

### 2.10 Intrinsic Fluorescence Spectroscopy

SPP or FSPP solution was prepared at a final concentration of 0.1 mg/mL using PBS as the solvent. Intrinsic fluorescence measurements were performed using a spectrofluorometer (F‐4700, Hitachi, Tokyo, Japan) with the following settings: excitation set at 280 nm, emission collected across 300–500 nm, both excitation and emission slits fixed at 5 nm, a scanning rate of 1200 nm/s, and a PMT voltage of 400 V (Yang, Kumar, et al. 2025).

### 2.11 Extrinsic Fluorescence Spectroscopy

SPP or FSPP solution (0.1 mg/mL) was mixed with 4 mM ANS solution at a ratio of 1:50 (v/v) and incubated in the dark for 15 min. Emission spectra were recorded from 400 to 650 nm at an excitation wavelength of 380 nm using a fluorescence spectrophotometer (F‐4700, Hitachi, Tokyo, Japan). Excitation and emission slit widths were both set at 5 nm, scanning speed was 1200 nm/s, and PMT voltage was 400 V (Yang, Kumar, et al. 2025).

### 2.12 Free Sulfhydryl Content

The quantification of free sulfhydryl groups was performed according to Wang et al. (2022). SPP or FSPP powder was suspended in 2 mg/mL glycine buffer. The resulting solution was reacted with 5 mg/mL Ellman's reagent in a 1:100 (v/v) proportion and allowed to stand at 25°C for 30 min. Absorbance was then recorded at 412 nm using a UV–visible spectrophotometer (UV‐1102, Tianmei Techcomp, Shanghai, China). The amount of free thiol groups was calculated using the following formula:

$$-\mathrm{SH}\left(\text{μmol}/g\right)=\frac{73.53\times A_{412}}{C}$$

where *A*_412_ is the absorbance at 412 nm and C is the concentration of the sample solution.

### 2.13 Surface Hydrophobicity

SPP or FSPP was prepared at concentrations ranging from 0.05 to 0.25 mg/mL by diluting with 0.01 M phosphate buffer (pH 7.0). A volume of 20 μL of ANS solution (8 mmol/L) was added to 4 mL of each diluted sample and allowed to react under dark conditions for 10 min. The fluorescence signal was recorded at excitation and emission wavelengths of 390 nm and 470 nm using a spectrofluorometer (F‐4700, Hitachi, Tokyo, Japan). The surface hydrophobicity (*H*₀) was estimated as the slope of the fluorescence–concentration relationship using a linear fitting approach, following the method reported (Wang et al. 2022).

### 2.14 Degree of Hydrolysis

Degree of hydrolysis (DH) was measured according to Yang, Kumar, et al. (2025) for six groups: SPP, FSPP, SPP‐SGF, FSPP‐SGF, SPP‐SIF, and FSPP‐SIF. Briefly, a 400 μL sample was mixed uniformly with 3 mL OPA reagent for 2 min, and absorbance was measured at 340 nm with a UV spectrophotometer. Standard and blank controls were replaced by serine standard solution and distilled water, respectively. DH was calculated using the equations:

$$\text{Serine}\;{\mathrm{NH}}_{2}=\frac{{\mathrm{OD}}_{\text{sample}}-{\mathrm{OD}}_{\text{blank}}}{{\mathrm{OD}}_{\text{stand}}-{\mathrm{OD}}_{\text{blank}}}\times 0.9516\;\text{mmol}/L\times \frac{1}{C}$$

$$\mathrm{DH}\left(\%\right)=\frac{\text{Serine}\;{\mathrm{NH}}_{2}-\beta }{\alpha \times h_{\mathrm{tot}}}\times 100\%$$

where *A*_standard_, *A*_sample_, *A*_blank_ are absorbances of standard, sample, and blank respectively; *C* is the concentration of the sample; *α* and *β* for SPP are 1.0 and 0.4, respectively; and h_tot_ is approximately 7.67.

### 2.15 Foaming Properties

A 20 mL sample solution (1 mg/mL) was placed into a 50 mL centrifuge tube, and initial volume was recorded. The solution was dispersed at 20,000 rpm for 2 min using a high‐speed homogenizer (T18, IKA, Germany), and the dispersed volume was immediately recorded. After standing at room temperature for 30 min, the volume was recorded again (Jiang et al. 2023). Foaming capacity (FC) and foaming stability (FS) were calculated as follows:

$$\mathrm{FC}\left(\%\right)=\frac{V_{0}}{V}\times 100$$

$$\mathrm{FS}\left(\%\right)=\frac{V_{30}}{V_{0}}\times 100$$

where *V* is the initial volume before dispersion, *V* is the volume immediately after dispersion, and *V*_30_ is the volume after standing for 30 min.

### 2.16 Emulsifying Properties

Soybean oil (1 mL) was mixed with sample solution (4 mL, 10 mg/mL) and dispersed at 10,000 rpm for 1 min using a high‐speed homogenizer (T18, IKA, Germany). Subsequently, 50 μL of the emulsion was mixed with 5 mL SDS solution (0.1%, w/v), and absorbance at 500 nm was measured. After standing at room temperature for 10 min, another 50 μL of the emulsion was similarly diluted and absorbance measured again (Jiang et al. 2023). The emulsifying assay was performed for six groups: SPP, FSPP, SPP‐SGF, FSPP‐SGF, SPP‐SIF, and FSPP‐SIF. Emulsifying activity index (EAI) and emulsifying stability index (ESI) were calculated as follows:

$$\mathrm{EAI}\left(m^{2}/g\right)=\frac{2T\times A_{0}\times N}{10000\times c\times \alpha \times L}$$

$$\mathrm{ESI}\left(\min\right)=\frac{A_{0}}{A_{0}-A_{10}}\times t$$

where *A*_0_ and *A*_10_ are absorbances at 500 nm immediately after preparation and after 10 min, respectively; *N* is the dilution factor; *c* is protein concentration; *α* is the volume fraction of oil; *L* is the path length; and *t* is the standing time of the emulsion.

### 2.17 DPPH Radical Scavenging Activity

Sample (1 μg/mL) from six groups (SPP, FSPP, SPP‐SGF, FSPP‐SGF, SPP‐SIF, or FSPP‐SIF) was combined with an equal volume of DPPH dissolved in ethanol (1:1, v/v), followed by a 30‐min reaction in the absence of light. The absorbance was recorded at 517 nm (*A*₁) (Huh 2022; Yang, Zhou, et al. 2025). The percentage of DPPH radical inhibition was determined using the formula below:

$$\text{DPPH scavenging activity}\;\left(\%\right)=\left(1-\frac{A_{1}-A_{2}}{A_{0}}\right)\times 100$$

where *A*_0_ is absorbance of control (distilled water instead of protein solution) and *A*_2_ is absorbance of ethanol control (95% ethanol instead of DPPH).

### 2.18 ABTS Radical Scavenging Activity

To prepare the ABTS radical solution, 7 mM ABTS was combined with 2.45 mM potassium persulfate in equal volumes (1:1, v/v) and allowed to react at room temperature in darkness for 12–16 h. The resulting stock was diluted in 0.01 M PBS buffer (pH 7.0) until its absorbance stabilized at 734 nm with a value of 0.70 ± 0.02, forming the ABTS working reagent. For the assay, 20 μL of test sample (1 μg/mL) from six groups (SPP, FSPP, SPP‐SGF, FSPP‐SGF, SPP‐SIF, or FSPP‐SIF) was introduced into 200 μL of the working reagent and incubated under light‐free conditions for 6 min. The absorbance was then measured at 734 nm (*A*₁) using a microplate spectrophotometer (Yang, Zhou, et al. 2025).

$$\text{ABTS scavenging activity}\;\left(\%\right)=\left(1-\frac{A_{1}-A_{2}}{A_{0}}\right)\times 100$$

where *A*_0_ is absorbance of control (distilled water instead of protein solution) and *A*_2_ is absorbance of PBS replacing ABTS.

### 2.19 Ferric Reducing Antioxidant Power (FRAP)

The FRAP analysis was conducted following the protocol provided in the Total Antioxidant Capacity Kit (Beyotime Biotechnology). In brief, 5 μL of the test sample was combined with 180 μL of FRAP reagent and incubated at 37°C for 5 min. The absorbance of the resulting solution was recorded at 593 nm. A calibration curve was generated using FeSO_4_ standard solutions (0.15–1.5 mM), and the antioxidant capacity was reported as FeSO_4_ equivalent concentrations. The assay was performed for six groups: SPP, FSPP, SPP‐SGF, FSPP‐SGF, SPP‐SIF, and FSPP‐SIF.

### 2.20 Hyaluronidase Inhibition Activity

A total of 0.5 mL of sample solution (SPP, FSPP, SPP‐SGF, FSPP‐SGF, SPP‐SIF, or FSPP‐SIF) was added to tubes A and B, and 0.5 mL of distilled water was added to tubes C and D. Tube A and C received 0.5 mL hyaluronidase solution, while tubes B and D received 0.5 mL acetate buffer. All tubes were incubated in a water bath at 37°C for 20 min. Then, 0.1 mL of 2.5 M CaCl_2_ was added to each tube, followed by incubation at 37°C for an additional 20 min. Subsequently, 2 mL of 0.4 mg/mL sodium hyaluronate was added to tubes A and C, while tubes B and D received 2 mL of acetate buffer. The reaction was maintained at 37°C for 40 min, then left at ambient temperature for 10 min. After that, 1 mL of acetylacetone reagent was introduced, and the tubes were placed in boiling water for 15 min, followed by rapid cooling in an ice bath of the same duration. After another 10‐min rest at room temperature, 1 mL of Ehrlich's reagent and 3 mL of anhydrous ethanol were added. The reaction mixtures were allowed to stand for 30 min, and the absorbance was recorded at 530 nm (Yang et al. 2024). The hyaluronidase inhibition rate was calculated as follows:

$$\text{Inhibition rate}\;\left(\%\right)=\frac{\left(A_{c}-A_{d}\right)-\left(A_{a}-A_{b}\right)}{A_{c}-A_{d}}\times 100$$

where *A*_a_ represents the absorbance of the sample solution (hyaluronidase + sample + sodium hyaluronate), *A*_b_ is the absorbance of the sample blank (acetate buffer + sample + acetate buffer), *A*_c_ is the absorbance of the control (hyaluronidase + deionized water + sodium hyaluronate), and *A*_d_ is the absorbance of the control blank (acetate buffer + deionized water + acetate buffer).

### 2.21 IgE‐Inhibition Activity

The IgE‐inhibition activity of SPP was determined as previously described (Tang et al. 2025; Zhao et al. 2022). Untreated SPP was used as the coating antigen, and samples from the six experimental groups were used as inhibitors in the competitive ELISA assay. A 96‐well plate was coated with 100 μL of protein solution per well and incubated at 4°C overnight. Wells were washed three times with PBST (150 μL/well, 2 min each) and blotted dry. Then, 250 μL of blocking buffer was added to each well and incubated at 37°C for 1 h. After washing and drying again, 50 μL of mixed serum (from silkworm‐allergic mice, Figure S2) and sample (as inhibitor) was added to each well and incubated at 37°C for 1 h. Wells were washed five times and blotted dry. Then, 100 μL of goat anti‐mouse IgE‐HRP secondary antibody was added and incubated at 37°C for 30 min. After washing, 100 μL of TMB substrate solution was added and incubated at 37°C for 15 min. The reaction was stopped by adding 50 μL of 2 mol/L sulfuric acid. Absorbance was measured at 450 nm. The IgE‐inhibition activity was calculated as follows:

$$\mathrm{IgE}-\text{inhibition activity}\left(\%\right)=\left(1-\frac{A_{0}}{A_{1}}\right)\times 100$$

where *A*_0_ = absorbance of the sample containing SPP; *A*_1_ = absorbance of the control sample without SPP.

### 2.22 Statistical Analysis

Each experiment was repeated three times. Data are presented as the mean with standard error of the mean (SEM). Graphical illustrations were produced using GraphPad Prism 9 (GraphPad Software Inc., San Diego, USA). Statistical evaluations were conducted through one‐way ANOVA, followed by Duncan's multiple range test using SPSS version 23 (IBM Corp., Chicago, IL, USA). Differences were regarded as significant when *p* values were below 0.05.

## 3 Results

### 3.1 Morphological Characterization of the Strain

**FIGURE 1:**
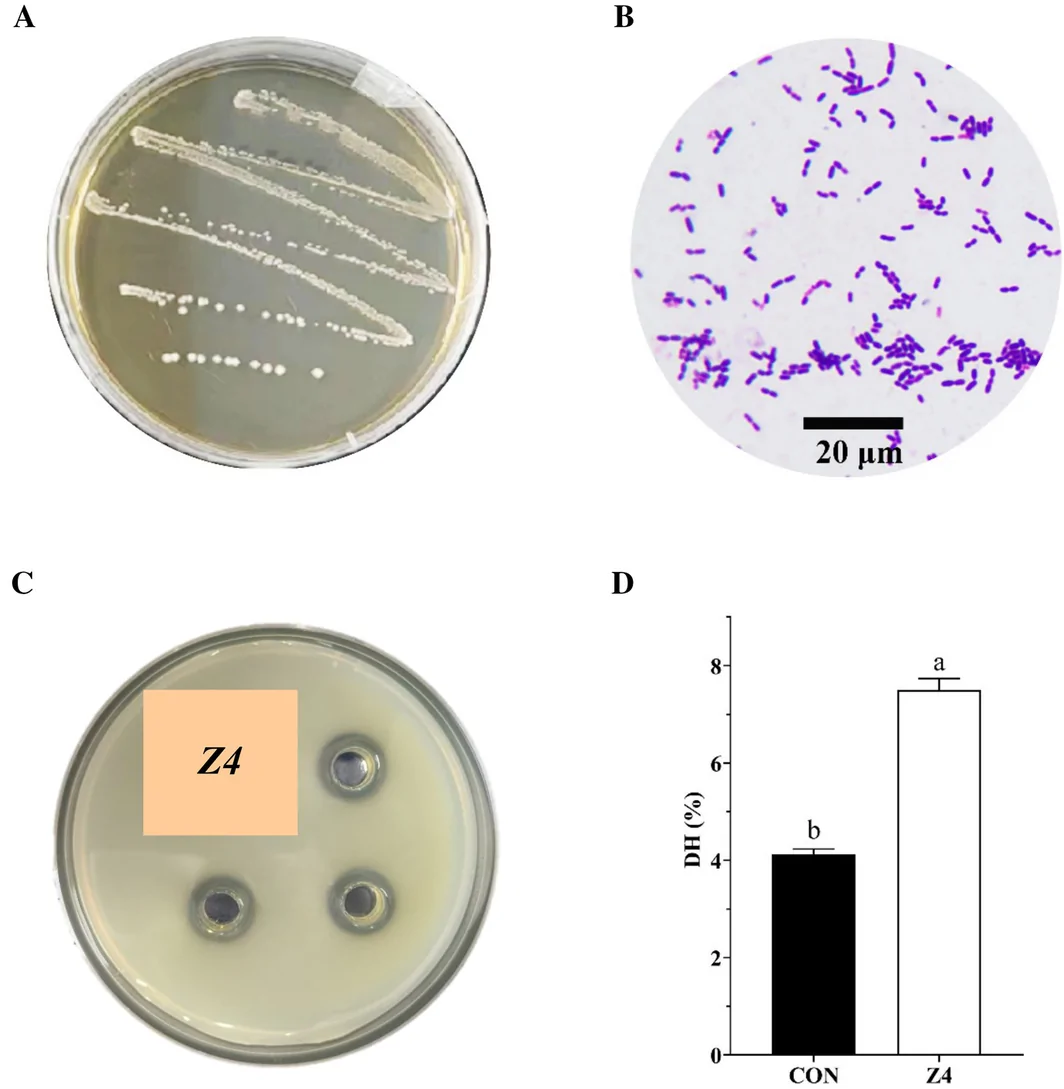
Morphological characteristics of *Lactiplantibacillus plantarum* Z4 and its hydrolysis activity on silkworm pupa protein. (A) Colony morphology of strain Z4; (B) Gram staining of strain Z4; (C) Protease activity of strain Z4; (D) Degree of hydrolysis. Data are presented as the mean ± standard error of the mean (SEM), *n* = 3. Different letters indicate significant difference (*p* < 0.05). CON, the non‐fermented SPP control, and Z4, SPP fermented with *Lactiplantibacillus plantarum* Z4.

The strain was isolated by streaking on an agar plate and examined using Gram staining. As shown in Figure 1, the isolated LAB formed milky white, smooth, and moist single colonies on the agar surface. Gram staining revealed blue‐colored cells, indicating that the strain is Gram‐positive. This result suggests the presence of a thick peptidoglycan‐rich cell wall, which is typical of LAB. The 16S rDNA gene sequence was subjected to homology analysis via the NCBI GenBank database. The sequence showed 100% similarity with known sequences of *Lactiplantibacillus plantarum* WILCCON 0030 (Figure S1). Therefore, the strain was identified as *Lactiplantibacillus plantarum* and designated as *L. plantarum* Z4.

### 3.2 Protease Activity of the Strain and Its Effect on the Degree of Hydrolysis of Silkworm Pupae Protein

The protease activity of *L. plantarum* Z4 was evaluated using the agar well diffusion method. As shown in Figure 1C, a clear hydrolysis zone was observed around the well containing the culture supernatant of strain Z4, indicating that this strain possessed proteolytic activity. The degree of hydrolysis (DH) of SPP after fermentation with *L. plantarum* Z4 is shown in Figure 1D. Compared with the unfermented control, fermentation with strain Z4 significantly increased the DH to 7.50% ± 0.38%. These results indicate that *L. plantarum* Z4 was able to hydrolyze protein substrates and may contribute to the hydrolysis of SPP during fermentation.

### 3.3 Effects of Fermentation on the Physicochemical Properties of Silkworm Pupae Protein

**FIGURE 2:**
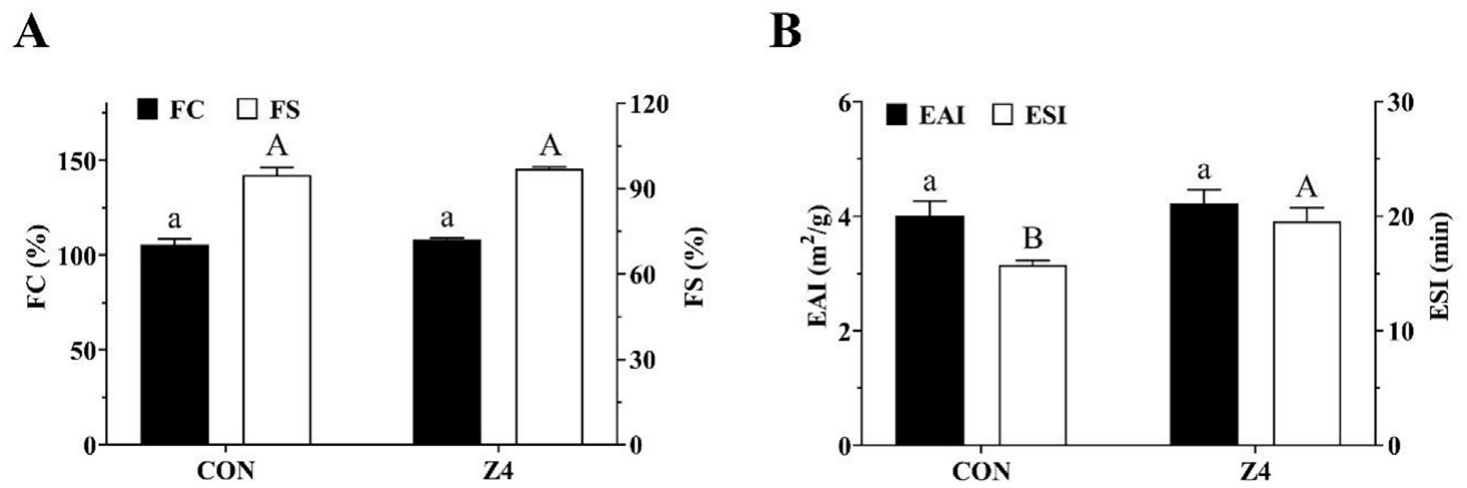
Changes in the physicochemical properties of silkworm pupa protein with fermentation. (A) FC and FS; (B) EAI and ESI. Data are presented as the mean ± standard error of the mean (SEM), *n* = 3. Different letters indicate significant difference (*p* < 0.05). CON represents the non‐fermented SPP control, and Z4 represents SPP fermented with *Lactiplantibacillus plantarum* Z4. EAI, emulsification activity index; ESI, emulsion stability index; FC, foam capacity; FS, foam stability.

FC reflects a protein's ability to form air‐liquid dispersions, while FS refers to the percentage of foam volume retained over time and is a key indicator of whipping stability in food systems (Figueroa‐González et al. 2022). In this study, no significant changes in the FS and FC of SPP after fermentation with *L. plantarum* Z4 were observed compared to the unfermented control (Figure 2A). The emulsifying properties of SPP were evaluated using the emulsifying activity index (EAI) and emulsifying stability index (ESI). As illustrated in Figure 2B, fermentation had no significant effect on EAI (*p* > 0.05), while ESI increased significantly, reaching 19.58 ± 1.19 min. These findings indicate that fermentation improved the emulsion stability of SPP without significantly affecting its initial emulsifying activity.

### 3.4 Effects of Fermentation on the Molecular Structure of Silkworm Pupae Protein

**FIGURE 3:**
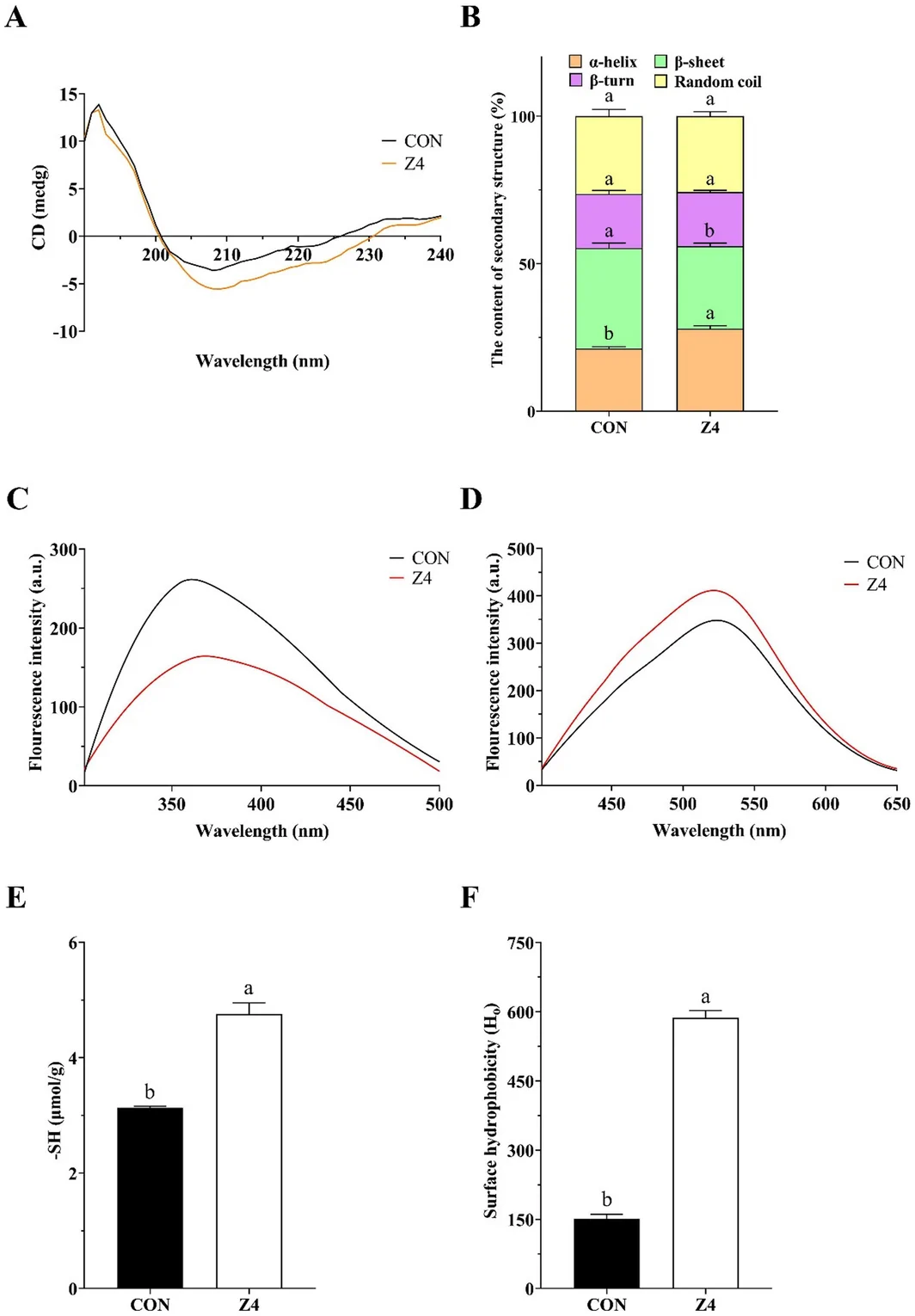
Changes in the molecular structure of silkworm pupa protein with fermentation. (A) Circular dichroism spectra; (B) Secondary structure changes of SPP; (C) Intrinsic fluorescence spectra; (D) Exogenous fluorescence spectra; (E) Content of free surface sulfhydryl groups; (F) Surface hydrophobicity. Data are presented as the mean ± standard error of the mean (SEM), *n* = 3. Different letters in (E, F) indicate significant difference (*p* < 0.05). CON, the non‐fermented SPP control, and Z4, SPP fermented with *Lactiplantibacillus plantarum* Z4.

Circular dichroism and fluorescence spectroscopy are effective techniques for analyzing the secondary and tertiary structures of proteins (Vinayashree and Vasu 2021; Wang, Zhou, et al. 2020). The secondary structure analysis of SPP is shown in Figure 3A,B. The untreated sample contained 21.67% ± 0.33% α‐helix, 34.00% ± 0.10% β‐sheet, 18.33% ± 0.67% β‐turn, and 26.33% ± 0.13% random coil. After fermentation with *L. plantarum* Z4, the α‐helix content increased significantly, while the β‐sheet content decreased significantly. The intrinsic fluorescence spectra (Figure 3C) revealed reduced fluorescence intensity and a red shift in the maximum emission wavelength from 361 nm to 369 nm after fermentation. In contrast, extrinsic fluorescence spectra using ANS (Figure 3D) showed increased fluorescence intensity and a blue shift in the maximum emission wavelength from 524 nm to 522 nm, suggesting increased exposure of hydrophobic regions. These results indicate that fermentation alters both secondary and tertiary structures of SPP.

Changes in sulfhydryl content are indicative of protein denaturation (Gao et al. 2023). The surface hydrophobicity index (*H*₀) reflects the relative content of exposed hydrophobic groups, which is a critical indicator of protein unfolding (Wang et al. 2022). As shown in Figure 3E, the free sulfhydryl content increased significantly from 3.13 ± 0.01 μmol/g in untreated protein to 4.75 ± 0.11 μmol/g in the fermented sample. Figure 3F shows that surface hydrophobicity also increased significantly, from 151.46 ± 5.24 to 586.47 ± 9.46. These results suggest that *L. plantarum* Z4 fermentation disrupted the tertiary structure of the protein, leading to molecular unfolding and greater exposure of hydrophobic residues.

### 3.5 Changes in Molecular Weight of Fermented Silkworm Pupae Protein After In Vitro Digestion

**FIGURE 4:**
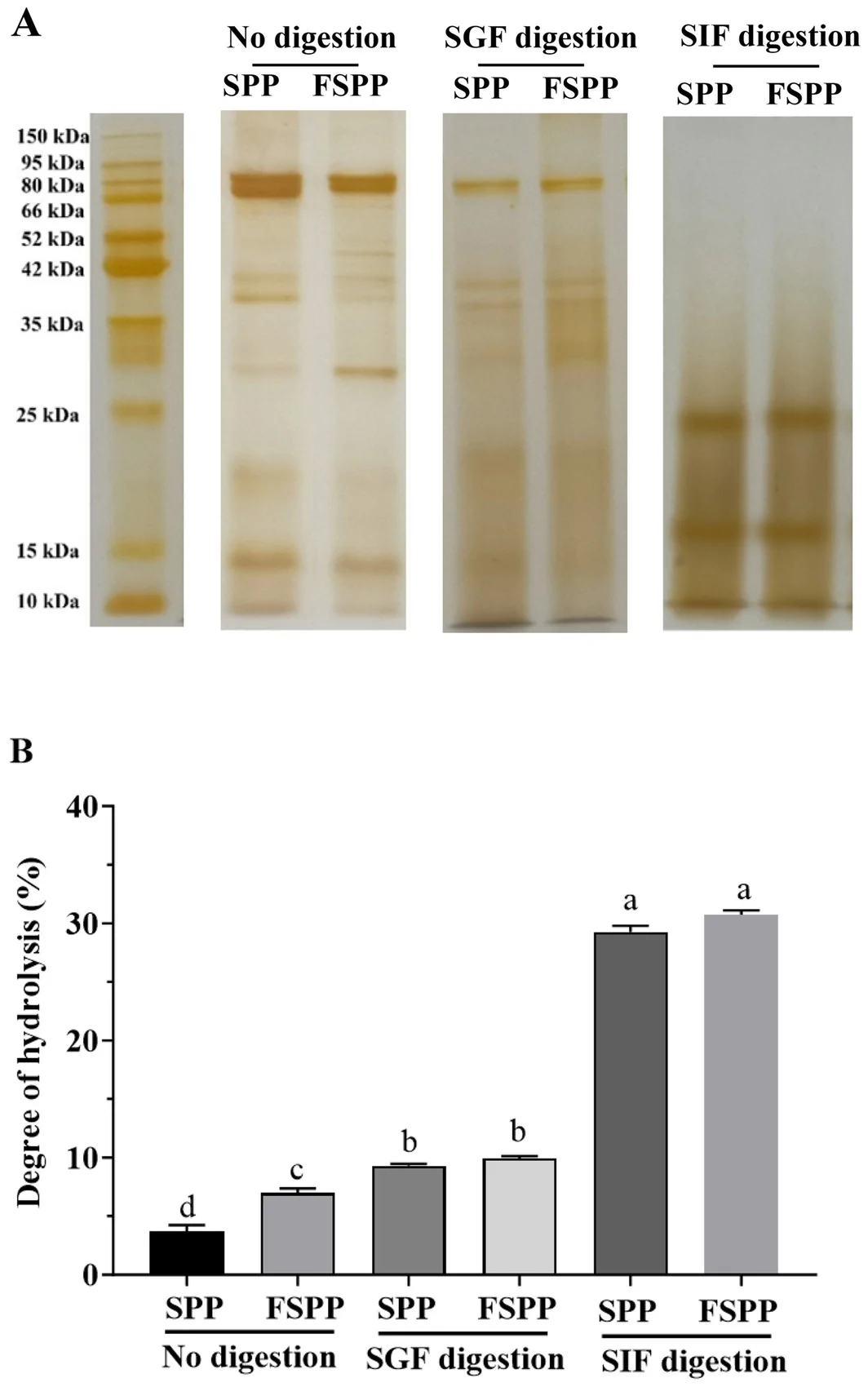
Changes in the molecular weight and degree of hydrolysis of silkworm pupa protein and silkworm pupa protein with fermentation before and after in vitro digestion (A, B). Different letters indicate significant difference (*p* < 0.05). FSPP, SPP fermented with *Lactiplantibacillus plantarum* Z4; SIF, simulated intestinal fluid digestion; SGF, simulated gastric fluid digestion; SPP, non‐fermented silkworm pupa protein.

To directly evaluate the effect of fermentation after gastrointestinal digestion, the digests of unfermented SPP and fermented FSPP were compared under the same simulated gastrointestinal conditions. SDS‐PAGE analysis showed that, after fermentation, the protein bands in the 66–80 kDa and 35–42 kDa regions became lighter, a new band appeared around 42–52 kDa, and the bands at 25–35 kDa became more intense (Figure 4A). The DH% of both SPP and FSPP increased significantly after SGF and SIF digestion, although fermentation increased DH before digestion. The highest DH values were observed after intestinal digestion, reaching approximately 35%–37%. These changes suggest that proteases produced by *L. plantarum* Z4 partially degraded SPP during fermentation. Although fermentation led to partial degradation of the native protein, simulated gastrointestinal digestion caused extensive hydrolysis of both SPP and FSPP, resulting in similar post‐digestion molecular weight distributions.

### 3.6 Effects of Fermentation and Digestion on the Antioxidant Activity of Silkworm Pupae Protein

**FIGURE 5:**
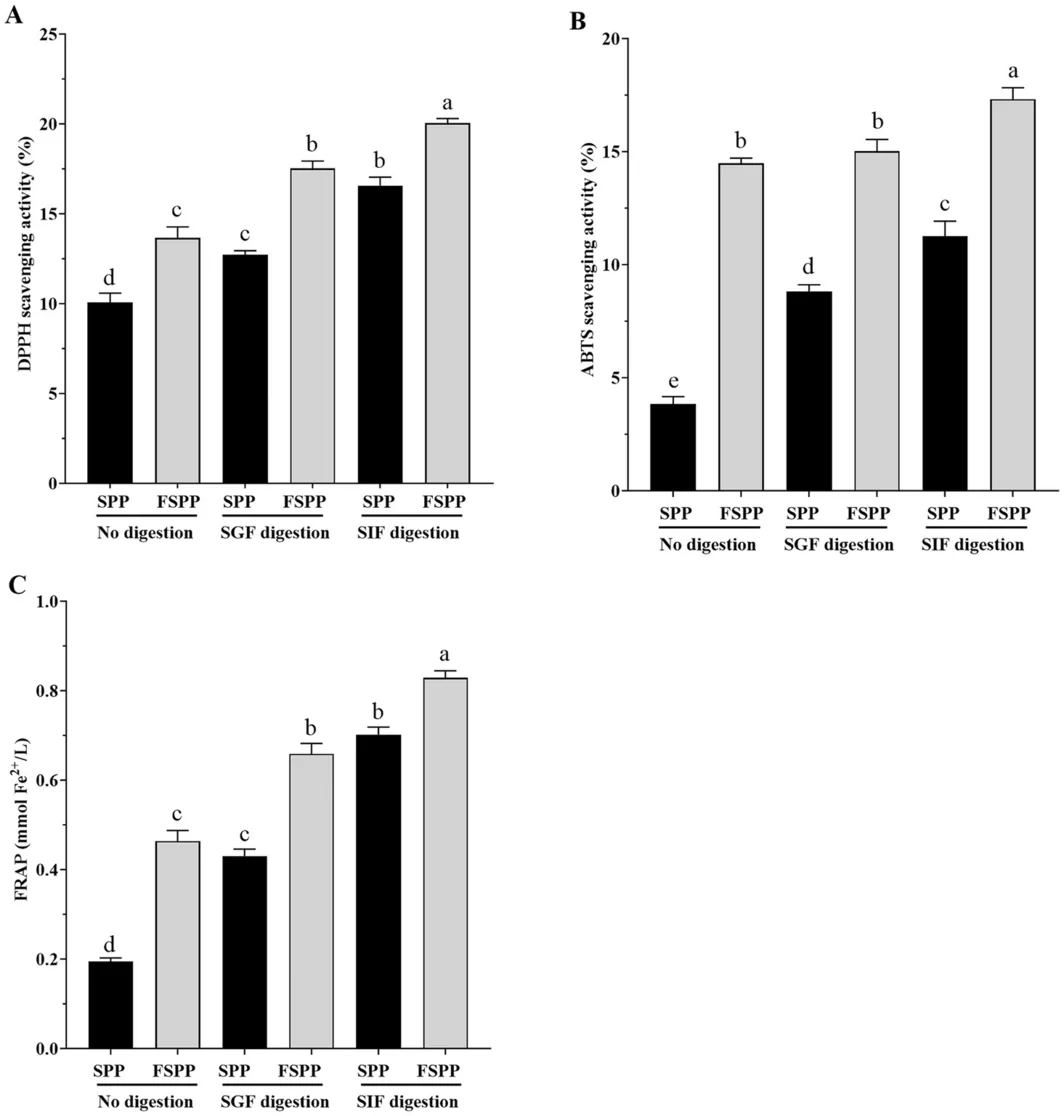
Changes in the antioxidant activity of silkworm pupa protein with fermentation before and after in vitro digestion. DPPH scavenging activity (A), ABTS scavenging activity (B), and FRAP (C) before and after in vitro digestion. Data are presented as the mean ± standard error of the mean (SEM), *n* = 3. Different letters indicate significant difference (*p* < 0.05). FSPP, SPP fermented with *Lactiplantibacillus plantarum* Z4; SIF, simulated intestinal fluid digestion; SGF, simulated gastric fluid digestion; SPP, non‐fermented silkworm pupa protein.

DPPH and ABTS radical‐scavenging assays are widely used to evaluate antioxidant activity (Ahmed et al. 2023; Zheng et al. 2022). As shown in Figure 5, fermentation significantly enhanced the antioxidant activity of SPP, as indicated by increased DPPH and ABTS radical scavenging activities and FRAP values. After in vitro gastrointestinal digestion, the antioxidant activities of both SPP and FSPP further increased, with the SIF digests showing the strongest activity. Under the same digestion conditions, FSPP exhibited higher antioxidant activity than the corresponding SPP group. These results indicate that gastrointestinal digestion enhanced antioxidant activity in both substrates, while Z4 fermentation provided an additional benefit beyond digestive proteolysis. Detailed numerical values and statistical annotations are provided in Table S1.

### 3.7 Effects of Fermentation and Digestion on the Allergenicity of Silkworm Pupae Protein

**FIGURE 6:**
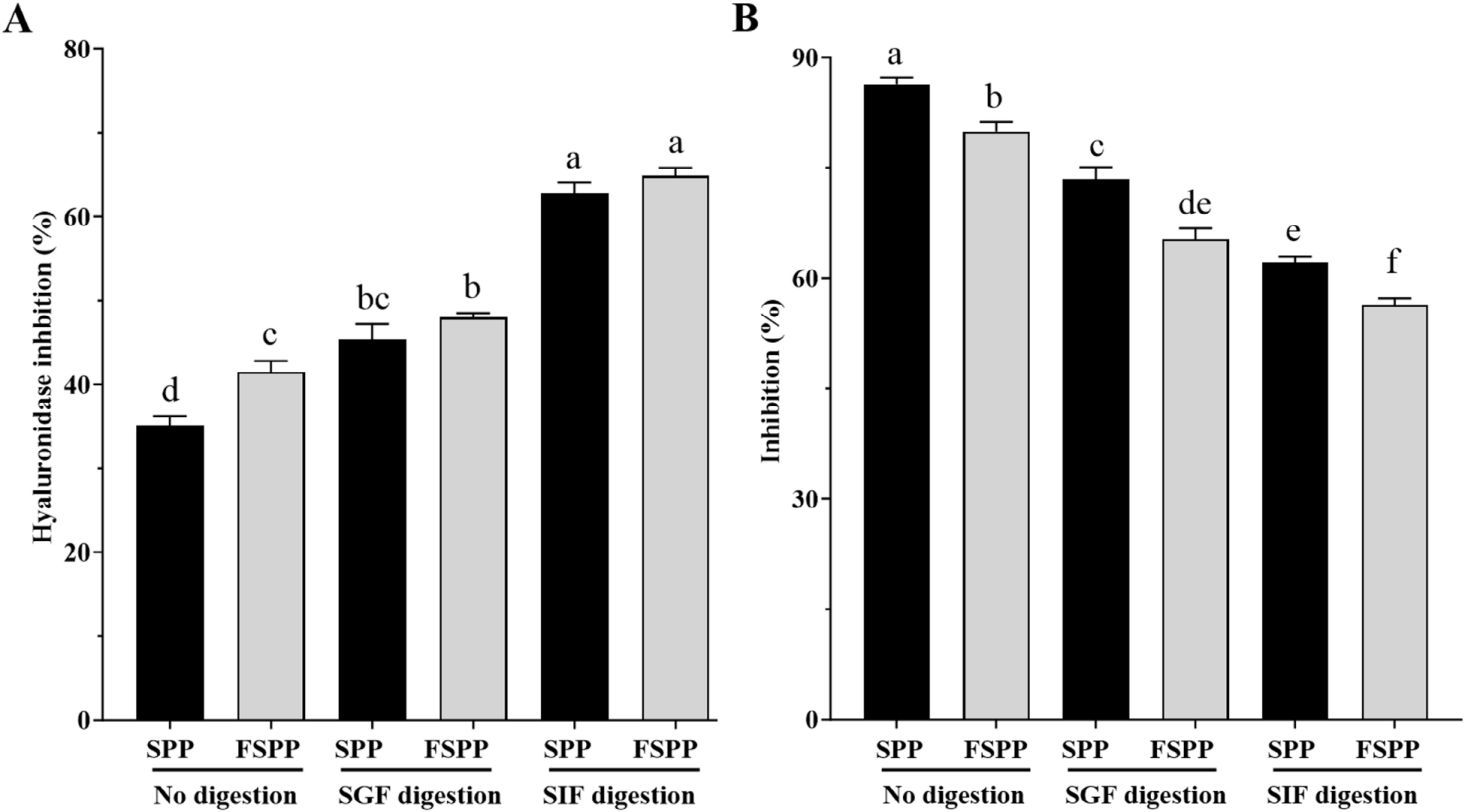
Changes in the allergenicity of silkworm pupa protein with fermentation before and after in vitro digestion. Hyaluronidase inhibition (A) and IgE‐inhibition activity (B) before and after in vitro digestion. Data are presented as the mean ± standard error of the mean (SEM), *n* = 3. Different letters indicate significant difference (*p* < 0.05). FSPP, SPP fermented with *Lactiplantibacillus plantarum* Z4; SIF, simulated intestinal fluid digestion; SGF, simulated gastric fluid digestion; SPP, non‐fermented silkworm pupa protein.

As shown in Figure 6, fermentation increased hyaluronidase inhibition and decreased IgE‐inhibition activity, indicating reduced allergenicity‐related responses of SPP. After in vitro gastrointestinal digestion, hyaluronidase inhibition further increased, whereas IgE‐inhibition activity further decreased in both SPP and FSPP. Under the same digestion conditions, FSPP generally showed a slight increase in hyaluronidase inhibition and significantly lower IgE‐inhibition activity than the corresponding SPP group. These results suggest that gastrointestinal digestion reduced allergenicity‐related responses in both substrates, while Z4 fermentation provided an additional benefit beyond digestive proteolysis. Detailed numerical values and statistical annotations are provided in Table S1.

## 4 Discussion

Recent studies have shown that the structure, functional properties, digestibility, and allergenicity of silkworm pupae protein can be modulated by different modification strategies. For example, chemical modifications such as phosphorylation, succinylation, deamidation, and glycosylation have been reported to alter the conformation, functional behavior, and IgE‐binding properties of SPP (Huang et al. 2025). Compared with these approaches, LAB fermentation represents a mild and food‐compatible modification strategy. In the present study, fermentation with *L. plantarum* Z4 induced structural changes in SPP and was associated with altered functional properties, enhanced antioxidant activity, and reduced allergenicity.

This study found that the DPPH and ABTS radical scavenging activities, as well as FRAP values, were significantly increased in SPP after fermentation. The enhancement in antioxidant activity may be attributed to the release of bioactive compounds during fermentation (Huo et al. 2023). It is reported that antioxidant activity increases in fermented protein products due to the presence of protein‐derived peptides (Dinkçi et al. 2023). Fermentation facilitates the release of peptides and amino acids with antioxidant properties from intact proteins, thereby enhancing overall antioxidant capacity (Abd Rashid et al. 2022). For instance, *Lactobacillus rhamnosus* fermentation increased the degree of hydrolysis and peptide yield from milk proteins (such as α‐casein, α‐lactalbumin, and β‐lactoglobulin), which contributed to elevated antioxidant activity (DPPH and ABTS radical scavenging) (Chawafambira et al. 2022). *Lactobacillus reuteri* WQ‐Y1 hydrolyzed casein and whey protein to release antioxidant peptides such as YLGYLEQLLR (from αS1‐casein), VKEAMAPK (from β‐casein), and YIPIQYVLSR (from κ‐casein), with VKEAMAPK showing the strongest activity (Cui et al. 2022). In insect study, fermentation of edible insect flours (mealworm and grasshopper) with *Lactococcus lactis* led to an increase in peptide diversity and significantly improved in vitro antioxidant activity (Mendoza‐Salazar et al. 2021). Therefore, it is likely that fermentation of SPP promotes the generation of antioxidant peptides, thereby enhancing its antioxidant potential. The DPPH, ABTS, and FRAP assays used in this study represent chemical radical‐scavenging potential and do not directly reflect cellular antioxidant activity. These assays were employed as preliminary indicators of antioxidant potential, while future studies will include cellular validation and peptide identification to further elucidate the underlying bioactive mechanisms.

In this study, in vitro gastrointestinal digestion further enhanced the antioxidant activity of SPP and FSPP, especially after intestinal digestion. Although the DH values and SDS‐PAGE profiles of digested SPP and FSPP were relatively similar, FSPP digests still exhibited stronger antioxidant activity. This indicates that the enhanced antioxidant activity was not fully explained by the overall hydrolysis degree or broad molecular weight distribution. Standard SDS‐PAGE mainly reflects relatively large protein fragments and provides limited information on small peptides, peptide sequences, or peptide abundance. Therefore, the higher antioxidant capacity of FSPP digests was more likely associated with differences in peptide composition, sequence characteristics, or structural features generated during fermentation‐assisted digestion. Z4 fermentation may act as a pre‐digestion modification step that alters subsequent enzymatic cleavage sites and promotes the release of more bioactive peptides during gastrointestinal digestion. Similar findings have been reported in edible insects, whose antioxidant activity increased after gastrointestinal digestion (Zielińska et al. 2017, 2018). In addition, antioxidant peptides derived from edible SPP hydrolysates have also been identified previously (Cermeño et al. 2022). Since peptides with similar molecular weights may still differ in bioactivity because of sequence and structural variation (Korhonen and Pihlanto 2006; Udenigwe and Aluko 2012), fermentation may have altered proteolytic cleavage during digestion and promoted the release of more bioactive peptides. However, the specific peptides responsible for these differences were not identified in the present study. Future HPLC or LC–MS/MS analysis is required to clarify the relationship between peptide composition and the enhanced antioxidant activity of FSPP digests.

Hyaluronic acid is a linear glycosaminoglycan composed of repeating units of d‐glucuronic acid and n‐acetyl‐d‐glucosamine. Due to its strong hydrophilicity and unique structural properties, it plays multiple roles in biological processes (Zhang et al. 2022). Hyaluronidase is an enzyme that degrades hyaluronic acid and is involved in allergic reactions by increasing the permeability of inflammatory cells. Therefore, hyaluronidase inhibition can help attenuate allergic symptoms by reducing tissue permeability and inflammatory infiltration and is commonly used as an in vitro indicator of anti‐allergic activity (Wang et al. 2021). In contrast, IgE‐binding reduction reflects a decrease in the allergenic potential of specific proteins, as IgE is responsible for initiating hypersensitivity reactions via mast cell activation (Pi et al. 2022). Although these two mechanisms target different phases of the allergic response, IgE‐binding acts at the sensitization phase, while hyaluronidase affects the effector phase—their combined modulation contributes to an overall anti‐allergic effect. In our study, the simultaneous reduction in both IgE‐binding and hyaluronidase activity suggests that fermentation and digestion not only decrease allergen recognition but may also reduce downstream allergic inflammation.

Similar results have been reported previously. LAB fermentation was shown to reduce the IgE‐binding capacity of peanut protein and wheat albumin (Fu et al. 2023; Pi et al. 2022), likely because protein degradation and structural modification masked or destroyed allergenic epitopes (Pi et al. 2022). For example, LAB fermentation of soy protein isolate significantly reduced IgE‐binding through peptide bond cleavage and conformational changes (Xing et al. 2024), while fermentation of soymilk with *Lactobacillus helveticus* disrupted higher order structures and damaged specific linear IgE‐binding epitopes, thereby lowering antigenicity (Lu et al. 2022).

In vitro digestion can further decrease allergenic potential. Previous studies showed that the IgG‐ and IgE‐binding capacities of allergenic proteins from almonds, pine nuts, peanuts, and eggs were significantly reduced after digestion (Takagi et al. 2005; Toomer et al. 2013; Yang, Kumar, et al. 2025). Consistent with these findings, the IgE‐inhibition activity of fermented SPP in our study was further reduced after in vitro digestion, in line with reports on fermented soymilk, wheat albumin, and soybean protein, where digestion further decreased IgE‐binding or enhanced IgE‐inhibition activity (Fu et al. 2023; Lu et al. 2022). These results suggest that fermentation may first disrupt allergenic epitopes, while subsequent digestion further degrades residual allergenic structures, ultimately leading to lower allergenic potential.

However, few studies have explored the relationship between fermented protein after digestion and hyaluronidase activity; evidence suggests that enzymatic hydrolysis can produce peptides with hyaluronidase inhibitory effects. For example, pepsin hydrolysates from salmon by‐products showed inhibitory activity against hyaluronidase (Wang et al. 2021). Comparative hydrolysis of salmon protein using different proteases revealed that pepsin hydrolysates exhibited the highest inhibition (50.27%), followed by trypsin (36.43%), alkaline protease (21.62%), and neutral protease (15.72%) (Wang, Siddanakoppalu, et al. 2020). The extent of hydrolysis can significantly influence peptide folding and conformation, which in turn affects their functional properties (Dai et al. 2025). This effect may be primarily attributed to protein hydrolysis, as higher degrees of hydrolysis generate smaller peptide fragments that may possess novel bioactivities. Although comparative LAB strains were not included in this study, these comparisons will be incorporated in future work to distinguish thermal from fermentation effects and to validate the strain‐specific advantages of *L. plantarum* Z4. Comparative fermentations using commercial *L. plantarum* and other LAB strains will be conducted to confirm the strain‐dependent variations in fermentation efficiency, functional enhancement, and allergenicity reduction. These efforts will further clarify the unique contribution of strain Z4 and strengthen the generalizability of the present findings.

## 5 Conclusion

Overall, Z4 fermentation directly modified the structure of SPP, enhanced its antioxidant activity, and reduced its allergenicity‐related responses before digestion. Gastrointestinal digestion further increased antioxidant activity and reduced allergenicity‐related responses in both SPP and FSPP. Under the same digestion conditions, FSPP digests generally showed greater antioxidant activity and lower IgE‐inhibition activity than the corresponding SPP digests, suggesting that Z4 fermentation provides additional functional benefits beyond digestive proteolysis and improves the digestive bioaccessibility of SPP‐derived components.

## Author Contributions

**Jing Yang:** conceptualization, funding acquisition, project administration, resources, writing – review and editing, supervision. **Shuling Zhou:** investigation, methodology, formal analysis, data curation. **Linqing Zhang:** investigation, data curation, writing – original draft. **Sutee Wangtueai:** writing – review and editing. **Jiajia Song:** writing – review and editing.

## Funding

This study was funded by Natural Science Foundation of Chongqing (Grant No. CSTB2024NSCQ‐MSX0745) and Graduate Research Innovation Project of Chongqing Municipal (CYS25623).

## Conflicts of Interest

The authors declare no conflicts of interest.

## Supporting information


**Figure S1:** Phylogenetic tree of strain Z4.
**Figure S2:** Hypersensitivity symptoms, and immunoglobulin level in serum. Hypersensitivity symptoms were scored within 1 h of the last challenge with SPP. (A) Each point represents an individual mouse. Serum from all mice was collected after euthanasia and used to measure the level of (B) SPP‐specific IgE tested by iELISA. Data are presented as the mean ± standard error of the mean (SEM) for each group. Control group (CON, *n* = 14 mice) and OVA‐induced allergy group (OVA, *n* = 14 mice). Superscript letter significant difference between CON and SPP (*p* < 0.05). SPP, silkworm pupae protein.
**Table S1:** Antioxidant activity and allergenicity‐related responses of SPP and FSPP before and after in vitro gastrointestinal digestion.

## Data Availability

The data that support the findings of this study are available from the corresponding author upon reasonable request.
