# Supplementary material for: Modification of Functional, Antioxidant, and Anti‐Allergic Properties of Fermented Silkworm Pupae Protein During In Vitro Gastrointestinal Digestion
Source: Food Sci Nutr. 2026 Aug 26;14(8):e72248. doi: 10.1002/fsn3.72248 (PMC13507931; doi:10.1002/fsn3.72248)
Supplement: Supplementary file 1 [file FSN3-14-e72248-s001.docx]

**Supplementary data**

**1.1 Identification of the strain by 16S rDNA gene sequencing**

Genomic DNA of the activated Z4 was isolated using a commercial bacterial DNA extraction kit. A 25 μL PCR system was employed with primers 27F and 1492R to amplify the target sequence. The resulting amplicons were sequenced by Sangon Biotech Co., Ltd. (Shanghai, China), and sequence similarity was verified through homology analysis using the NCBI BLAST platform.

**1.2 Preparation of specific polyclonal antibodies to silkworm pupae protein**

SPF-grade female BALB/c mice (4 weeks old) were housed under controlled environmental conditions: temperature of 20-25 °C, relative humidity of 45-60%, and a 12-hour light/dark cycle. The Animal Care Committee of Chongqing Technology and Business University approved these studies. Mice were provided with standard laboratory chow and water. After a one-week acclimation period, animals were randomly assigned into separate cages. A total of 28 mice were divided into two groups: a control group (n = 14) and a silkworm pupae protein-induced allergy group (n = 14). Mice in the sensitized group received intraperitoneal injections on days 0, 7, 14, 21, and 28 with 300 µL of a sensitization mixture containing 150 µL of untreated silkworm pupae protein solution (300 µg/mL) and 150 µL of aluminum hydroxide adjuvant. The control group received the same volume (300 µL) of a mixture containing PBS and aluminum hydroxide without silkworm pupae protein. On day 35, mice in the sensitized group were orally challenged with 200 µL of untreated silkworm pupae protein solution at a concentration of 100 mg/mL to elicit an allergic response (Dai et al., 2023).

**1.3 Scoring of allergic symptoms in mice**

Allergic symptoms induced by silkworm pupae protein were evaluated according to the method described previously (Liu et al., 2023). Clinical symptoms were observed and scored within 1 h after oral challenge. Allergic behaviors were observed and allergy scores were recorded: 0=no symptoms; 1=repeated scratching and rubbing of the head around the nose; 2=puffiness around the eyes and nose, diarrhea, upright wall, decreased activity, and increased respiratory rate; 3=wheezing, labored breathing, cyanosis of the mouth and tail; 4= tremors and convulsions, and 5= death. Mice were quickly euthanized by cervical dislocation. Following observation, blood samples were collected and centrifuged at 3000 × *g* for 10 min at 4 °C, and the resulting serum was aliquoted and stored at -80 °C for further analysis.

**1.4 *IgE-binding ability analysis by indirect Enzyme-linked immunosorbent assay (iELISA)***

A 96-well microtiter plate was coated with 100 µL of untreated silkworm pupae protein solution (50 μg/mL) per well and incubated overnight at 4 °C under sealed conditions. The next day, the coating solution was discarded, and the wells were washed three times with 150 µL/well of PBST (0.05% Tween-20 in PBS) with gentle shaking for 2 min each time, followed by blotting to remove residual liquid. The wells were then blocked with 250 µL/well of blocking buffer and incubated at 37 °C for 1 h. After blocking, the wells were emptied, washed three more times with PBST as previously described, and blotted dry. Subsequently, 50 µL of serum from silkworm pupae protein-sensitized mice was added to each well and incubated at 37 °C for 1 h. After incubation, the wells were washed five times with PBST and blotted dry. Then, 100 µL of horseradish peroxidase (HRP)-conjugated goat anti-mouse IgE secondary antibody was added to each well and incubated at 37 °C for 30 min. After three additional PBST washes, 100 µL of TMB substrate solution was added to each well and the plate was incubated at 37 °C for 15 min in the dark. The reaction was terminated by adding 50 µL of 2 mol/L sulfuric acid, followed by gentle shaking. The absorbance was measured at 450 nm using a microplate reader. The serum dilution that produced an absorbance value between 0.7 and 0.8 was selected for subsequent experiments (Hu et al., 2024).

**Fig. S1**. Phylogenetic tree of strain Z4.

**A**

**B**

**Fig. S2.** Hypersensitivity symptoms, and immunoglobulin level in serum. Hypersensitivity symptoms were scored within 1 h of the last challenge with SPP. (A) Each point represents an individual mouse. Serum from all mice was collected after euthanasia and used to measure the level of (B) SPP-specific IgE tested by iELISA. Data are presented as the mean ± standard error of the mean (SEM) for each group. Control group (CON, n = 14 mice) and OVA-induced allergy group (OVA, n = 14 mice). Superscript letter significant difference between CON and SPP (*p* < 0.05). SPP, silkworm pupae protein.

Table S1. Antioxidant activity and allergenicity-related responses of SPP and FSPP before and after *in vitro* gastrointestinal digestion.

| **Group** | **DPPH radical scavenging activity (%)** | **ABTS radical scavenging activity (%)** | **FRAP (mmol Fe²⁺/L)** | **Hyaluronidase inhibition (%)** | **IgE-inhibition activity (%)** |
| --- | --- | --- | --- | --- | --- |
| **SPP** | 10.06 ± 0.52**^d^** | 3.84 ± 0.32**^e^** | 0.19 ± 0.01**^d^** | 35.11 ± 1.14**^d^** | 86.36 ± 0.85**^a^** |
| **SPP-SGF** | 12.71 ± 0.23**^c^** | 8.23 ± 0.28**^d^** | 0.43 ± 0.02**^c^** | 45.37 ± 1.88**^bc^** | 73.51 ± 1.57**^c^** |
| **SPP-SIF** | 16.57 ± 0.47**^b^** | 11.26 ± 0.66**^c^** | 0.70 ± 0.02**^b^** | 62.77 ± 1.29**^a^** | 63.59 ± 0.83**^e^** |
| **FSPP** | 13.65 ± 0.61**^c^** | 14.48 ± 0.24**^b^** | 0.46 ± 0.02**^c^** | 41.48 ± 1.32**^c^** | 79.94 ± 1.33**^b^** |
| **FSPP-SGF** | 17.52 ± 0.41**^b^** | 15.02 ± 0.52**^b^** | 0.66 ± 0.02**^b^** | 48.00 ± 0.51**^b^** | 65.27 ± 1.52**^de^** |
| **FSPP-SIF** | 20.04 ± 0.26**^a^** | 17.31 ± 0.52**^a^** | 0.83 ± 0.02**^a^** | 64.89 ± 0.92**^a^** | 56.30 ± 0.96**^f^** |

Data are presented as the mean ± SEM, n = 3. Different superscript letters within the same column indicate significant differences among groups (*p* < 0.05). SPP, non-fermented silkworm pupae protein; FSPP, SPP fermented with *L. plantarum* Z4; SGF, simulated gastric fluid digestion; SIF, simulated intestinal fluid digestion.
